# A Gentle Introduction to the Comparison Between Null Hypothesis Testing and Bayesian Analysis: Reanalysis of Two Randomized Controlled Trials

**DOI:** 10.2196/10873

**Published:** 2018-10-24

**Authors:** Marcus Bendtsen

**Affiliations:** 1 Division of Community Medicine Department of Medical and Health Sciences Linköping University Linköping Sweden

**Keywords:** null hypothesis testing, Bayesian analysis, randomized controlled trials, Bayes theorem, randomized controlled trials as topic

## Abstract

The debate on the use and misuse of *P* values has risen and fallen throughout their almost century-long existence in scientific discovery. Over the past few years, the debate has again received front-page attention, particularly through the public reminder by the American Statistical Association on how *P* values should be used and interpreted. At the core of the issue lies a fault in the way that scientific evidence is dichotomized and research is subsequently reported, and this fault is exacerbated by researchers giving license to statistical models to do scientific inference. This paper highlights a different approach to handling the evidence collected during a randomized controlled trial, one that does not dichotomize, but rather reports the evidence collected. Through the use of a coin flipping experiment and reanalysis of real-world data, the traditional approach of testing null hypothesis significance is contrasted with a Bayesian approach. This paper is meant to be understood by those who rely on statistical models to draw conclusions from data, but are not statisticians and may therefore not be able to grasp the debate that is primarily led by statisticians.

## Introduction

### Background

In response to a growing concern that claims of new discoveries as a result of scientific studies are becoming less and less credible, Benjamin et al [1] (signed by 71 authors) recommended that the threshold used to determine statistical significance should be reduced from the conventional .05 to .005. To do so, they claim, would immediately improve the reproducibility of scientific research in many fields. The authors acknowledge that any choice of threshold is arbitrary and that it incorporates a trade-off between false-positive and false-negative findings, yet they partially justified their choice of .005 by saying that it would reduce the false-positive rate to levels that they judge to be reasonable. In their concluding remarks, the authors pointed out that the proposed threshold should not be used to reject findings with *P* values between .005 and .05, but they should rather be labelled as suggestive evidence. Regarding this recommendation, Amrhein and Greenland [2] commented that, while this trichotomization may be better than the prevailing dichotomization into what is significant or not, it does not solve the issues of *P* hacking, selective reporting, and publication bias. Rather, the authors argued, it will only inflate these problems. Scientific conclusions should be based on multiple studies, and to allow for an unbiased and valid synthesis of the literature, all results must be published, regardless of *P* values. Furthermore, Amrhein and Greenland [2] pointed out that inference from a mathematical model cannot become “the truth” just because it passes some predefined threshold, and thus the authors suggested removing statistical significance completely.

Not only does the conventional null hypothesis testing using a threshold value of .05 constitute a requirement for publication, but as McShane et al [3] pointed out, it also constitutes a requirement for the results to be taken seriously. If the null hypothesis is not rejected, then researchers are stuck between two conditions in terms of conclusions, and are often far too eager to make a misinterpretation of no effect, since the null hypothesis was not rejected. Due to this, McShane et al [3] argued, considerations of the study design and quality of the data collected, prior and related evidence, plausibility of the mechanism that is investigated, novelty of the finding, real-world benefits and costs, etc, are only considered after *P* values have been checked, and if the threshold is broken, then little concern is given to these other factors. In this sense, whether or not statistical significance has been achieved has been given a superior standing over other equally important factors, and, since *P* values’ main purpose has been to check for such significance, they too have been given elevated status. McShane et al [3] proposed putting *P* values on the same level as all other factors, thus abandoning statistical significance as an arbiter of truth, and treating *P* values as a continuous measure. The authors further argued that letting null hypothesis testing guide scientific discovery does not make sense, since the hypothesis tested is exactly no effect, which can never happen in an experimental setting and is in general very implausible (that an intervention has exactly no effect, whether it be positive or negative, is in most cases impossible). Thus, it is often forgotten that *P* values are calculated assuming a world in which the intervention has exactly no effect, but the probability of this world occurring is essentially zero. It should be emphasized that a *P* value is a mathematically correct and good answer to how likely a result is given a particular null hypothesis and may in some cases be a good enough approximation, but this in and of itself should not be a crucial factor for publication. McShane et al [3] support a holistic view of the evidence, in which all relevant factors are taken into consideration when interpreting statistical analyses, and this holistic view should also be adopted by journal editors and reviewers.

Voices have been raised over the past few years against the use and misuse of *P* values, perhaps most notably in a formal statement from the American Statistical Association clarifying widely agreed-upon principles underlying the proper use of *P* values [4], the banning of *P* values from the journal *Basic and Applied Social Psychology* [5], and Nuzzo’s splendid summary in *Nature* [6]. McShane and Gal’s [7] article is a fascinating read regarding the alarmingly widespread misinterpretation of *P* values and null hypothesis testing among both researchers who are not primarily statisticians and those who are. The ensuing discussion also gives an interesting insight into this problem and potential solutions [8-12].

### Objective

This paper does not repeat the evidence put forward regarding the misinterpretation of *P* values, but instead contrasts the conventional null hypothesis and *P* value approach with that of a Bayesian analysis approach. The Bayesian approach taken is not in any sense novel, but has rather been proposed and used before; see Browne et al [13], Goodman and Sladky [14], Krushke [15], Morris et al [16], Spiegelhalter et al [17], and Wijeysundera et al [18]. However, as has been pointed out before [18], it is necessary to include nonstatisticians in the process of moving to a Bayesian approach. Therefore, this paper aims to inform those who routinely use null hypothesis testing and *P* values in the reporting of their research results, but who may not be responsible for running the analysis and may therefore find the discussion led by statisticians hard to grasp. Throughout, we attempt to give just enough understanding of the involved concepts so as to avoid too much technical detail, but at the same time we do not trivialize to the point where the discussion again becomes abstract. We begin by refreshing the reader’s memory regarding probability distributions, since they play such a crucial role in statistical analyses, and then we use a coin flipping experiment to describe and contrast the conventional approach and the Bayesian approach. At this point, we turn our attention to real-world data, reanalyzing 2 randomized controlled trials. Finally, with a better understanding of the two approaches, we revisit the discussion outlined here.

## Probability Distributions

As mentioned in the introduction, we do not attempt to offer an exhaustive discussion about the finer details of any mathematical aspects unless absolutely necessary. There is, however, no escaping the fact that it is necessary to understand, at least at a conceptual level, the notion of a probability distribution.

If we randomly pick a person from the general population, then we cannot, before we make our pick, possibly know their height. But we can do better than just saying that we know nothing about this person’s height, since we do have an idea about people’s heights in general. For instance, we know that the height cannot be negative and that it is unlikely to be more than 250 cm. Science requires us to reason in a systematic fashion, and for us to do so we need to express our knowledge about people’s heights mathematically. Commonly this is done by assigning a probability distribution to our random person’s height. A probability distribution is a purely mathematical construct that can tell us how likely different heights are relative to one another. So, it could tell us how likely it is that the person we pick will be between 160 and 180 cm tall, or how likely it is that the person will be taller than 150 cm. There are infinitely many probability distributions to pick from, and which one we use is our choice: we pick one that encodes our knowledge about people’s heights. It should not be forgotten that probability distributions are mathematical constructs that help us create a systematic picture of the real world, but they make no claim to represent any truth about the real world.

**Figure 1:**
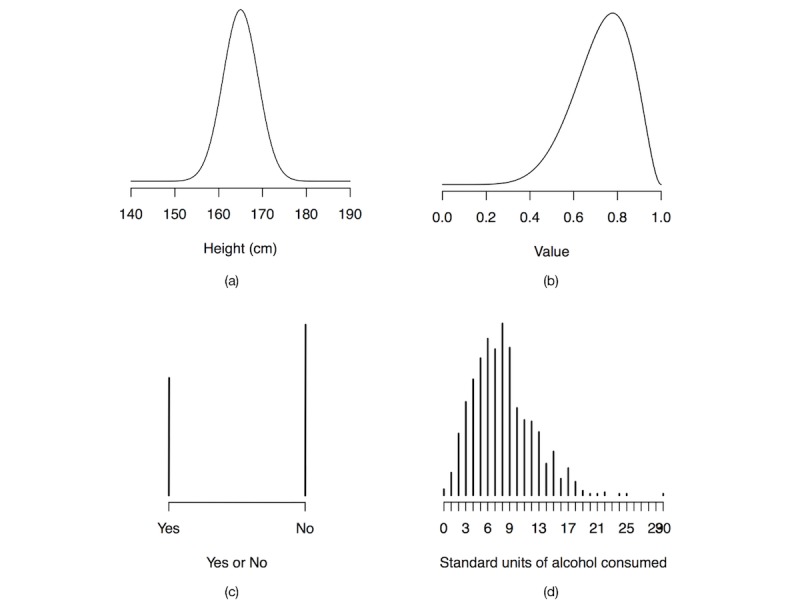
(a) A normal distribution with a mean value of 165 cm and standard deviation of 4 cm. (b) A beta distribution with shape parameters 8 and 3. (c) A Bernoulli distribution with *q*=0.6. (d) A negative binomial distribution with parameters 8 and 0.5 (failures and success probability).

For our purposes, we can think of probability distributions as shapes rather than mathematical equations. For instance, Figure 1 (part a) depicts a probability distribution for the height of a randomly picked person from the general population. We are here assuming that the distribution of heights can be represented using a *normal* distribution with a *mean* value of 165 cm and a *standard deviation* of 4 cm (this may not map perfectly to the real world; however, it is the choice that we have made). The mean and standard deviation are *parameters* of the normal distribution that tell us where we should center the distribution and how wide it is. Think of parameters as fine-tuning our choice of probability distribution—that is, we first picked the normal distribution and then we fined-tuned it using the parameters mean and standard deviation. Looking at Figure 1 (part a), we can see that values close to 165 cm are more likely than values further away, since the shape is higher around 165 cm. Figure 1 (part b) depicts a different distribution, known as the *beta* distribution. The beta distribution assigns probabilities only to values between 0 and 1; it would not be a good choice for modelling height, since we do not expect a person’s height to be confined between 0 and 1, but the beta distribution can be used to model uncertainty about other problems. The beta has two parameters known as shape, so we can also fine-tune the beta distribution for our purposes.

In some cases, we have a finite number of outcomes. For instance, in a randomized controlled trial, we may have responses from participants to a yes-or-no question (eg, “Have you smoked any cigarettes the past week: yes or no”). In such cases, we can use a *Bernoulli* distribution that works over only two possible outcomes (a normal or beta distribution would not make sense here). A Bernoulli distribution has a parameter that we call *q* that tells us how likely it is that a participant will respond “no.” Figure 1 (part c) depicts a Bernoulli distribution with the *q* parameter set to 0.6 (ie, we are encoding that there is a 60% chance that a participant answered “no”). As we can see, the shape is no longer a curve, but rather consists of bars that show how likely the outcomes are relative to one another. If we were investigating the number of whole standard units of alcohol consumed per week by a population, then we would have more than two possible outcomes, all greater than or equal to 0. In such cases, we could potentially use a *negative binomial* distribution, depicted in Figure 1 (part d), where we can see that there are more than two outcomes over which the distribution is defined.

The point to remember is that probability distributions allow us to encode uncertainty about quantities that we do not know the exact value of. For instance, if somebody asks what the height is of a randomly picked person off the street, we do not have to say “I do not know,” but might instead answer “The height will follow a normal distribution with mean 165 cm and standard deviation 4 cm.” There is a myriad of different probability distributions to pick from, and they all have different parameters that we can fine-tune to make sure that they encode our knowledge correctly. To understand most of this paper, we can think of probability distributions as shapes, just like the ones depicted in Figure 1 (a through d).

## Null Hypothesis Significance Testing and Bayesian Analysis of a Coin Flipping Experiment

We contrast the prevalent approach of null hypothesis significance testing (NHST) with a Bayesian analysis approach. For this comparison to be as simple as possible, in this section we use a classic experiment that we are all familiar with: flipping a coin and recording whether it lands heads or tails. Later, we compare the two approaches by reanalyzing 2 randomized controlled trials. However, to understand how the two approaches fundamentally differ, we begin by using a simple experiment and model.

### Data and Model

Our experiment consists of flipping a coin 1000 times. We shall assume that the coin landed with heads up 540 times out of these 1000 flips. These are the data that we have collected: 540 heads and 460 tails. We would like to know whether the coin that we have used is fair—that is, whether the coin was manufactured in such a way that it is equally likely to get heads or tails when we flip it.

To encode and communicate the uncertainty about the outcome of flipping a coin, it is common to say that the outcome follows a Bernoulli distribution. We recall from the previous section on probability distributions that the Bernoulli distribution works over two possible outcomes (here we have heads or tails) and that it has a parameter *q* that in this experiment represents the probability of heads. We formally state our model as Equation 1:


*coin flip ~ Bernoulli(q)*

*q = ?*

The squiggly line should be read as “follows,” so that the model expresses the story “a coin flip follows a Bernoulli distribution with parameter *q* and the value of *q* is unknown.” Do not overanalyze Equation 1, as all it does is communicate to others that we believe that when we flip our coin there are two possible outcomes (Bernoulli) and that there is a probability *q* that our coin will land with heads up (but we do not know the value of *q* yet). It should be stressed that this is just a mathematical model of a coin flip, and there is nothing *true* about it. In fact, the model is actually wrong, since there is at least theoretically a third outcome, that the coin lands on edge standing straight up. A further infinite number of outcomes can be generated by considering the rotation of the coin.

We have our data (540 heads over 1000 flips) and our model in Equation 1, and our analysis should now revolve around the value of *q*. We therefore in the next two sections employ first an NHST approach and then a Bayesian approach to the analysis of *q*.

### Null Hypothesis Significance Testing

When taking the NHST approach, we believe that there exists a fixed *population value* for *q* in Equation 1. In the coin flip experiment, it is easy to think of this population value as tied to some physical property of the coin. While one should avoid the word *true* when it comes to statistics, since all our inferences are based on a model that we have picked, we may think of this population value as the true value of *q*. In experiments involving a human study population, such as university students or office employees, the population value can be thought of as the value of *q* for the entire population. In most studies, we have only a sample of the entire population, in which case we cannot possibly know the population value for *q*. Note that it is not always clear what we mean by the population value, since study populations are often large to infinite in size, and sometimes the population is not very well defined (university students is a quite loosely defined group that changes from year to year). Nevertheless, the population value has a central role in the NHST approach.

#### Maximum Likelihood Estimator

We begin by considering the *maximum likelihood estimator* for *q*. This estimator is the value of *q* for which the likelihood of the data that we have collected is maximized. To decrypt what we mean by this, we can intuitively think of the maximum likelihood estimator as outlined in Textbox 1.

Returning to our original experiment, the maximum likelihood estimator for *q* would therefore be 540/1000=0.54 (recall that we had recorded 540 heads). It should, however, not come as a surprise that, if we went back and restarted the experiment and flipped the same coin 1000 times again, we would get a different outcome, for instance, 525 heads. This would then imply a different maximum likelihood estimate of 525/1000=0.525. In this way, we can see the maximum likelihood estimator as a proxy for the data that we have collected, a single number that summarizes information about the data with respect to the model.

As a side note, because of the rather simple model that we are employing (Equation 1), the maximum likelihood estimator was easy to calculate. It is, however, not always so, and for other models it may be necessary to apply optimization techniques to identify the maximum likelihood estimator. Most of us need not to worry about these details; we can assume that we can get a maximum likelihood estimator for most models.

Having calculated the maximum likelihood estimator, the next step is to consider a *sampling distribution*.

**Calculating the maximum likelihood estimator for *q*.**

Assume that we had recorded only 10 heads out of 1000 coin flips and that somebody suggests that the value of *q* should be 0.9, or a 90% probability of heads. Most of us would disagree and say that if *q*=0.9 then recording only 10 heads out of 1000 coin flips would be very unlikely. Another value might then be suggested, such as *q*=0.4, but we would still object, saying that 10 heads out of 1000 coin flips with a coin that is supposed to give 40% heads seems unlikely. So for which value of *q* would 10 heads in 1000 coin flips be most likely? It turns out that in this case it is trivial to calculate: 10/1000=0.01. So the value for *q* that makes 10 heads out of 1000 coin flips most likely is 1%, and this is the maximum likelihood estimator.

#### Sampling Distribution

From the discussion about maximum likelihood estimators, we concluded that, if we were to restart the coin flipping experiment, we could (even if we used the same coin) get a different number of heads. This would also then result in a different maximum likelihood estimator. Let us extend this line of thought and consider redoing the experiment thousands and thousands of times. What could we say about the maximum likelihood estimators that we would calculate for each one of these experiments? Just like we cannot know the exact height of a randomly picked person off the street before we actually pick and measure them, we cannot know which maximum likelihood estimator we will get next time we run the experiment. But this does not mean that we are totally unknowledgeable about the outcome: just like there is a distribution of heights, there is a distribution of maximum likelihood estimators. Theory tells us that this distribution is centered at the population value, and that it can be approximated by a normal distribution (at least when sample sizes are big enough). It is this distribution that is referred to as the sampling distribution. Each time we redo our coin flipping experiment, we get a maximum likelihood estimate that follows the sampling distribution (just like picking a person from the general population gives us a measurement of their height that follows the height distribution).

In our discussion about probability distributions, we mentioned that a normal distribution has two parameters: mean and standard deviation. The mean decides where the distribution is centered and the standard deviation decides how wide it is. We have established that the sampling distribution can be approximated by a normal distribution and that its mean (ie, its center) is the population value. Using our original data (540 heads over 1000 flips), we can use theoretical results to calculate an approximation of the standard deviation of the sampling distribution (often referred to as the standard *error*). In our case this value is approximately 0.0158.

Let us recapitulate. Given the data that we have collected (540 heads over 1000 flips), and the model that we have chosen (Equation 1), we can calculate a maximum likelihood estimate for *q* (540/1000=0.54), which follows a normal sampling distribution that is centered at the population value for *q* and has a standard deviation of 0.0158. Using this information, we can return to our original question: is the coin that we have flipped fair? To answer this, we turn to the practice of using hypothesis testing and *P* values.

#### Hypothesis Testing and P Values

We have previously stated that we wish to investigate whether the coin that we flipped was fair, and therefore our *null hypothesis* states that the population value for *q* is 0.5 (a *q* value of 0.5 means that there is a 50% probability of heads). If the null hypothesis fails to hold, we will instead accept an alternative hypothesis, which states that the population value for *q* is not 0.5 (ie, the coin is not fair).

**Figure 2:**
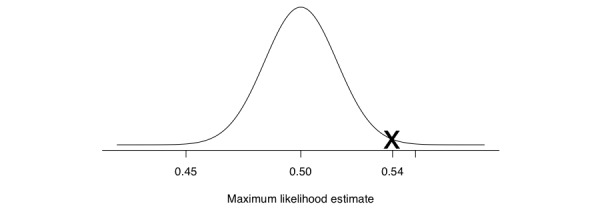
Sampling distribution of *q* under the null hypothesis that the population value is 0.5. The X marks the maximum likelihood estimate of the coin flip experiment (540 heads over 1000 flips).

We now enter a hypothetical world in which we assume that the null hypothesis is true. This is a key concept: we are going to analyze our data in a world in which we know that the null hypothesis is true, and therefore the population value for *q* is known to be 0.5. Now recall that the sampling distribution that we defined previously was centered at the population value of *q*, but back then we did not know the population value. Now we know the mean and standard deviation of the sampling distribution and we can draw a representation of it. In Figure 2 we can see the sampling distribution for our maximum likelihood estimates (remember, just like people’s heights have a distribution, so do maximum likelihood estimates). Additionally, in Figure 2 we have marked the maximum likelihood estimate that we calculated using the data that we collected in our experiment (540 heads over 1000 flips). Our maximum likelihood estimate is quite far out to the right, and such a value seems unlikely under this specific sampling distribution (the curve is low). Now recall that we said that the maximum likelihood estimate is a proxy for our data, a summary that we can use instead of the 1000 flips. This therefore tells us that the data that we have collected are quite unlikely, given that the population value is 0.5. But how unlikely? Enter *P* values. You sometimes hear people explain *P* values as “the probability of seeing these data or more extreme.” It is sometimes hard to understand what is meant by more extreme data. What they are actually trying to say is “seeing this maximum likelihood estimate or higher” (or lower depending on which side of the center we are looking at).

Because we have approximated the sampling distribution using a normal distribution, it is easy to calculate the probability of a maximum likelihood estimator of 0.54 or more extreme given a mean value of 0.5 and a standard deviation of 0.0158. It turns out that this probability is approximately 0.0057, and we must multiply this value by 2 because we wish to do 2-sided tests (this has to do with the fact that we arbitrarily decided to do our calculations based on heads rather than tails). Therefore, our final *P* value is .0114.

Since this *P* value is less than the conventional threshold of .05, we say that the data that we have collected are so unlikely given the null hypothesis that we reject the null hypothesis and accept the alternative hypothesis. This is referred to as statistical significance. However, given the .005 threshold proposed by Benjamin et al [1], we cannot reject the null hypothesis and we would therefore not be able to say anything about the fairness of the coin.

To summarize, we enter a hypothetical world in which our null hypothesis is true, and if the data that we have collected seem unlikely or absurd in this world, then we reject the hypothesized world. But it does not say much about which world is the *true* world—that is, it does not say much about the population value. To narrow in on the population value, it is common to also report confidence intervals, which we turn to next.

#### Confidence Intervals

Our maximum likelihood estimate is only 1 draw from the sampling distribution, so it does not tell us what the population value of *q* is. Recall that we are assuming that the population value of *q* is a fixed value, something that represents the entire population. One way of informing us about the location of the population value is to create what is known as confidence intervals.

Using a threshold of .05, we have already concluded that we will reject the null hypothesis that *q*=0.5, since the *P* value (.0114) was less than this threshold. We could increase our null hypothesis a bit, say to 0.501 rather than 0.5, and redo our hypothesis test as before. We would get a new *P* value of .0136, which would also lead to a rejection at the .05 threshold. But if we continue to increase the value of our null hypothesis, we would end up with a hypothesis that we cannot reject. This value is the lower limit of the confidence interval. Likewise, we can start from above our maximum likelihood estimate of 0.54 and find the largest value for our null hypothesis that cannot be rejected. The lowest and highest values that cannot be rejected are the confidence limits, and any hypothesis between these 2 limits cannot be rejected using the data that we have collected at the .05 threshold. In our coin flipping experiment, these limits are 0.509 and 0.571; thus, no hypotheses between these 2 values could be rejected given our data (540 heads over 1000 flips). Because we have chosen a threshold value of .05, these confidence intervals are known as 95% confidence intervals.

It would be nice if we could say that the population value of *q* lies within these 2 limits with 95% probability. But we cannot do so, unfortunately. Recall that if we could go back in time and redo the experiment, we would get a different maximum likelihood estimate; this means that we would also get a different set of confidence limits. What we can say, although it is very cumbersome, is that out of all the 95% confidence intervals that would be created by redoing the experiment, the population value for *q* will lie within them in 95% of the cases. If this sounds confusing, then you are in good company; most researchers tend to forget or misunderstand this.

#### Summary

This ends our introduction to the NHST approach. While we have attempted a high-level overview, we have nevertheless covered some central concepts that are necessary to keep in mind when applying this approach:

- The population value is a fixed value that we want to investigate.
- We collect data and compute maximum likelihood estimates for our model’s parameters.
- We construct a sampling distribution (a distribution over maximum likelihood estimates).
- We hypothesize a population value, entering a world in which we assume that we know its true value.
- If, in the hypothesized world, the data are unlikely given some threshold, then we reject the null hypothesis—that is, we reject this world.
- We create confidence intervals, which tell us which hypotheses we cannot reject, and enable us to say something about the location of the population value (although this information might be very vague).

### Bayesian

We have seen how the NHST approach focuses on understanding how likely the data gathered are given a sampling distribution and different hypothesized population values of *q*. The outcome of the analysis is information about which hypotheses we can and cannot refute given a predefined threshold. The Bayesian approach, however, asks the more direct question “How probable is every value of *q*?” There are an infinite number of *q* ’s that we could pick, and the Bayesian wants to know how probable each one of them is, given the data that we have. The Bayesian approach does not rely on repeated experimentation to create a sampling distribution, but rather looks only at the probability for every *q* given the data that we have collected. What we receive by requesting this information is not a single value, such as the maximum likelihood estimate, but an entire distribution over all possible values of *q*.

The Bayesian philosophy is to begin with a belief about the quantity of interest (in our case, *q*), and then look at the data that have been collected and revise one’s belief in light of the data. This is why words such as *updating* or *learning* are often used to describe the Bayesian approach, as we update our beliefs given the data, or alternatively learn something new from the data. To make this philosophy more formal, we rely on three concepts: prior distributions, data likelihood, and posterior distributions. We discuss these three in order, using the same coin flipping experiment as before.

#### Prior Distributions

When a quantity is unknown to us, such as *q* has been when we have been flipping coins, the Bayesian approach is to assign to this quantity a prior distribution. This prior distribution encodes our uncertainty about *q* before we analyze the data that we have collected. The keyword here is *before* (ie, prior). Just like the outcome of flipping a coin is unknown to us, so is the value of *q*. Our solution for describing the uncertainty about the coin flip was to say that it follows a Bernoulli distribution, and our solution for describing our uncertainty about *q* is to say that it also follows some distribution. There is a bit of harmony here, as we are not treating unknown quantities differently: as soon as the value of something is unknown to us, we say that it follows a distribution, regardless of whether it is data or parameters.

**Figure 3:**
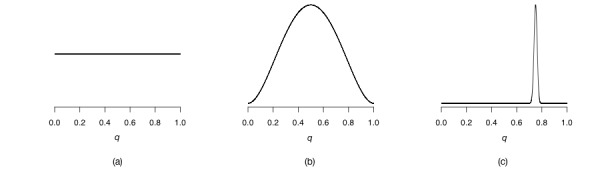
(a) Uniform prior distribution (flat prior). (b) A prior distribution that encodes that fair coins are more likely. (c) A prior distribution that encodes that biased coins are more likely.

Recall that we have at our disposal many distributions that we can use to describe uncertainty: we have already encountered the normal, beta, Bernoulli, and negative binomial distributions. We also have the option of saying that we think that each value of *q* is equally likely before we analyze the data: we then say that *q* follows a uniform distribution. This is sometimes referred to as a *flat* prior, since the shape of the probability distribution is a flat line. Figure 3 (parts a through part c) presents three examples of different priors that we could choose: Figure 3 (part a) depicts a flat prior—that is, it assigns the same probability to every possible value of *q*. Figure 3 (part b) depicts a prior that says that we believe the coin to be fair before we start flipping it, so we assign more probability to *q* values around 0.5, but we are still assigning quite a bit of probability to all other values (the shape is wide). Finally, Figure 3 (part c) says that we believe the coin to be biased, assigning almost all probability to *q* values around 0.75 (the shape is very narrow).

When starting out with Bayesian analysis, it may seem like one would always want to pick a flat prior, like the one depicted in Figure 3 (part a). At first glance, this might seem like an objective choice, as there is no bias toward any specific value, and the NHST approach essentially takes this stance. However, this is not as objective as one might first think, and we shall return to this point in our discussion. Sometimes it may be beneficial to pick priors that enable analysis, for instance, if the number of potential participants in a study is very low, expert information may be encoded into the prior allowing for the analysis to still output useful results; please see Goodman and Sladky [14] and Morris et al [16] for examples. Another case is when we have many covariates to choose from, but we wish to include only the relevant ones for the outcome in our model [19,20].

If we decide to use a flat prior for our coin flipping experiment, then we extend our model to express that before we collect any data we believe all values for *q* to be equally likely (Equation 2):


*coin flip ~ Bernoulli(q)*

*q ~ uniform(0,1)*

The equation now reads “We believe that coin flips follow a Bernoulli distribution and that the probability of heads is *q*. We also believe that *q* is equally likely to take on any value between 0 and 1.” Recall that only values between 0 and 1 make sense for *q*, since it represents the probability for heads, so we cannot have negative probabilities, nor probabilities above 1. Compare this with our original model in Equation 1, where we said that *q* was completely unknown; using priors forces us to be more specific and explicit about what we mean when we say that something is unknown.

That is all we need to say about priors for the moment. They make sure that we express the uncertainty about all unknown values up front before we start the analysis.

#### Data Likelihood

Akin to what we were calculating before, during the NHST discussion, the data likelihood tells us how remarkable the data that we have collected are given different values of *q*. If we propose that *q*=0.5, then we can calculate the probability that we would collect 540 heads over 1000 flips with this proposed value. We can make this calculation because we have chosen a model for our experiment (we chose a Bernoulli distribution); if we had no model, then we could not make any of these calculations. Intuitively, we would expect that if we had instead proposed that *q*=0.1, then the data should be less likely than when *q*=0.5, since we have collected 540 heads over 1000 flips.

**Figure 4:**
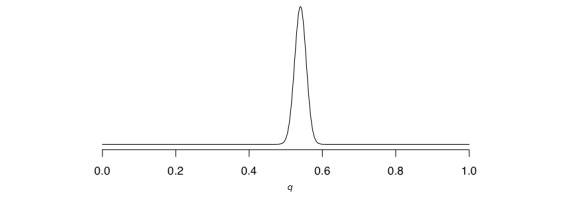
Data likelihood for different values of *q* in the coin flipping experiment.

If we continued this reasoning for every possible value of *q*, then we could draw a shape that tells us how likely the data that we have collected are for different values of *q*. Such a shape is drawn in Figure 4 for our coin flipping experiment. As we can see, this follows our intuition that the data are more likely given values for *q* around 0.5 compared with values around 0.1.

The shape in Figure 4 can be thought of as the data likelihood given our model. It tells us how remarkable the data that we have collected are given different values for *q*.

#### Posterior Distributions

The prior distribution encodes what we believe about *q* before we take into consideration any data, and the data likelihood tells us how remarkable the data that we have collected are given different values of *q*. But what we really care about is what we believe about *q after* we have taken into consideration the data. This is encoded in the *posterior* distribution, and it is the posterior distribution that is the answer to the Bayesian question “How probable is every value of *q*?”

The posterior distribution is a distribution just like all the others we have seen in this paper. It is calculated using Bayes’ theorem. This theorem is a consequence of basic probability theory and named after famous statistician Reverend Thomas Bayes. Equation 3 is the simplified version. The theorem states that the posterior distribution can be computed by multiplying the data likelihood by the prior distribution.


*posterior ∝ likelihood × prior*

**Figure 5:**
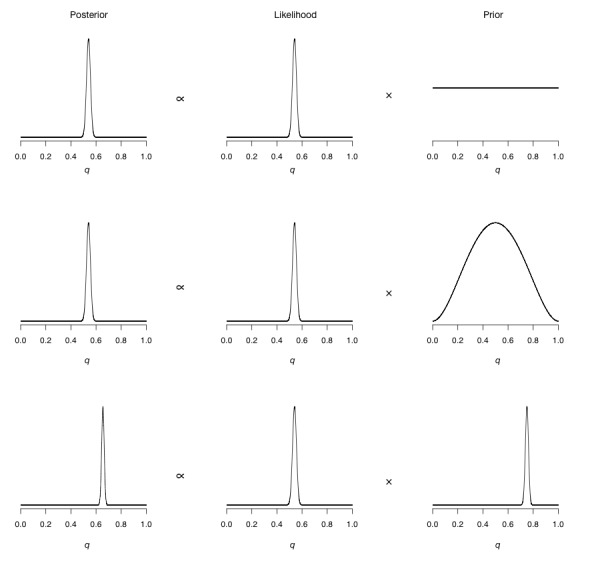
Three examples of the use of Bayes’ theorem for the coin flipping experiment. Top row: posterior distribution when using a uniform prior distribution (flat prior). Middle row: posterior distribution when using a weak prior distribution that makes fair coins more likely. Bottom row: posterior distribution when using a strong prior distribution that makes biased coins more likely.

Rather than discussing this in terms of numbers, let us instead do this graphically, as we have been thinking of distributions as shapes rather than as equations. What we will be doing is essentially multiplying the priors that we depicted in Figure 3 a through 3c by the data likelihood depicted in Figure 4. In Figure 5, we can see Bayes’ theorem in action for our coin flipping experiment. In each row we have a single use of the theorem. The top row shows us the result when using a flat prior, which is multiplied by the data likelihood to get a posterior. The second row shows us the use of the theorem with a prior that assigns more probability of the coin being fair, but does still allow for the entire range of possible *q* values (sometimes known as a weakly informative prior). The third and final row shows us the use of the theorem when we have a prior that very strongly believes that the coin is biased, using a very narrow prior around the value of 0.75 (note that this prior does not say that it is impossible that *q* can be 0.2, for instance; it just assigns a very small prior to this value of *q*). It is the column marked *Posterior* that is of interest at the moment. As we can see, the first 2 rows seem to have the same posterior: *q* values between 0.5 and 0.6 seem to be most probable according to these 2 rows. This is not a mistake; a common theme in Bayesian analysis is that once we have enough data the prior gets overwhelmed by the sheer amount of data. The prior that we picked for row number 2 assigned enough probability to all values of *q* that the data could easily overwhelm it, but not so for row number 3. In row number 3, we can see that the posterior distribution is shifted to the right; here values above 0.6 and less than 0.7 are more probable. The prior in the third row so strongly believed that the coin was biased that the data could not overwhelm it; thus, the entire posterior distribution is shifted toward the prior.

What we are saying is that the posterior probability of a value of *q* should take into consideration how likely this value was before we collected the data (the prior) but also how remarkable the data that we collected are under this value of *q*. So, for instance, if we were to collect 540 heads over 1000 flips and propose a *q* value of 0.01 (ie, a 1% chance of heads), then the posterior distribution for *q*=0.01 would be very low, since collecting 540 heads over 1000 flips when the probability of heads is only 1% is very unlikely. But proposing values around *q*=0.5 and *q*=0.6 should generate higher posterior probabilities, since 540 heads over 1000 flips is a lot more likely for such values.

We need not worry about the details of exactly how these calculations are done, but remember that Bayes’ theorem is remarkably simple: the posterior is computed by multiplying the data likelihood by the prior distribution. Also note that the output of the Bayesian analysis is the posterior distribution—that is, a distribution over the parameter of interest (in this case *q*) after we have taken into consideration the data that we collected.

#### Analysis of the Coin Flipping Experiment

**Figure 6:**
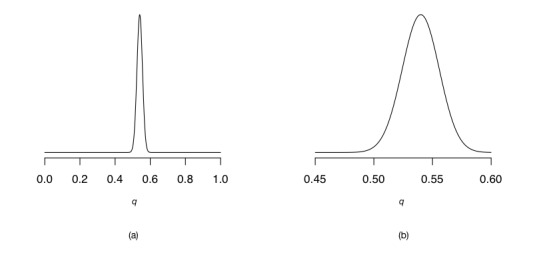
(a) Posterior distribution of *q* after observing 540 heads given 1000 flips (0≤*q* ≤1). (b) Posterior distribution of *q* after observing 540 heads given 1000 flips (0.45≤*q* ≤0.6).

Figure 6 (part a) depicts the posterior distribution over *q* for our coin flipping experiment using a flat prior (this is the same as the top row in Figure 5). We have zoomed in on *q* values between 0.45 and 0.6 in Figure 6 (part b). What does the posterior distribution tell us? Just by looking at it, we can see that it is quite unlikely that *q*=0.2. However, it is important to note that we are not ruling out this case; it is still entirely possible that *q*=0.2, but given the coin flips that we have made it is logically less likely that *q*=0.2 compared with, say, 0.5. It seems that the most likely value of *q* relative to all others is around 0.54 (see the zoomed-in distribution in Figure 6, part b). It is, however, crucial to note that the result of the Bayesian analysis is not a single value such as 0.2, 0.5, 0.54, or 0.6, but rather the entire posterior distribution over *q*.

Once we have a posterior distribution over our parameter *q*, we can ask scientific questions about how probable different values of *q* are. We initially stated that we wished to investigate whether the coin was fair or not. A coin that is biased to resulting in more heads than tails would imply a *q* value greater than 0.5 (ie, there is a greater than 50% chance of heads), so we may ask “What is the probability that *q* is greater than 0.5?” The answer is given by the posterior distribution, and in this case it is approximately 99%. To see this, look at Figure 6 (part b) again and color the entire area underneath the curve above 0.5. As you can see, the area that you have colored far outweighs the area you have not. The story that we tell is, therefore, that “We flipped a coin 1000 times and 540 times it landed heads. There is a 99% probability that the coin is biased toward showing more heads than tails.”

In this case, it is hard to argue against the coin being biased because there was a 99% probability of it being so, but what is the conclusion if the probability was 60%? In the real-world data analysis that we will conduct, we shall encounter such a case and we shall therefore defer this discussion. Essentially, it ties into what McShane et al [3] referenced as neglected factors; that is, what are the real-world costs and benefits of the finding, how novel is this finding, given previous studies what does this finding tell us, etc. Definitive dichotomous conclusions belong to the NHST approach, not the Bayesian.

#### Summary

The Bayesian approach begins by assigning prior probability distributions to unknown quantities, extending our models to also encode uncertainty about the parameters. Using the likelihood of the data, the prior is updated using Bayes’ theorem, resulting in a posterior distribution. The posterior distribution encodes the uncertainty about the model’s parameters after we have taken the data into consideration.

We will now leave the fictitious coin flipping experiment that we have been treating here and instead focus on real-world data collected during randomized controlled trials. We will defer any contrasting between the NHST approach and the Bayesian approach described here to the general discussion section.

## Analysis of Real-World Data

So far we have been using a rather trivial coin flipping example to illustrate the differences between the NHST and the Bayesian approaches. In this section, we instead look at data that were collected during 2 randomized controlled trials and complete a Bayesian analysis of the 2 trials in order to compare with the NHST analyses that have been published previously [21,22]. We shall look at the evaluation of a smoking cessation program and an alcohol consumption reduction program, both targeted at university students in Sweden and consisting of text messages sent to participants’ mobile phones. We shall not delve into the details of the interventions, but will rather refer to them as the NEXit (for smoking) and AMADEUS (for alcohol) trials.

We begin by analyzing the NEXit trial: first, we describe the statistical model; second, we account for the NHST analysis already conducted; third, we conduct the new Bayesian analysis; and fourth, we discuss the outcome. We shall follow the same structure for the AMADEUS trial.

### NEXit Trial

The NEXit trial was a single-blind, 2-arm, randomized controlled trial conducted between October 2014 and April 2015. Participants were daily or weekly smokers willing to set a quit date within 1 month of enrollment. Almost all college and university students in Sweden were contacted via email and invited to participate. Willing participants who fulfilled the inclusion criteria were randomly allocated to 2 groups: an intervention group that received the novel intervention and a control group that were asked to quit smoking on their own. The primary outcome measure was prolonged abstinence, defined as not having smoked more than 5 cigarettes during the past 8 weeks, and a 4-week point prevalence of complete smoking cessation (ie, no cigarettes smoked during the past 4 weeks). We shall not reanalyze any secondary outcomes.

#### Statistical Model

Both primary outcome measures in the NEXit trial were binary: participants responded either yes or no to the questions regarding prolonged abstinence and point prevalence. Just like in the coin flipping experiment, we are faced with two possible outcomes, and we do not know which outcome we will get if we randomly pick a NEXit participant. To reason systematically, we can say that the primary outcome measures in the NEXit trial follow a Bernoulli distribution with parameter *q*, where *q* represents the probability of a participant responding that they have not smoked (we treat each outcome measure separately). However, we would like to go a bit further and define a model that allows for different *q* values depending on whether a participant belongs to the control group or the intervention group, allowing us to contrast the difference between these *q* values. In a sense we wish to find 2 coins, 1 for each group, and compare whether one coin is more or less biased than the other.

The canonical way of modelling the narrative just given is to use what is known as logistic regression. We will avoid delving deeper into the details of this model, since the analysis here can be understood without them. What is important to note is that the quantity that is normally investigated is the *odds ratio* between the intervention group and the control group. The odds ratio is the odds of not smoking in the intervention group divided by the odds of not smoking in the control group. This quantity is convenient because it tells us by how much we should multiply the odds in the control group to get the odds in the intervention group. Thus, if the odds ratio is 1, then there is no effect, since you would take the control group’s odds and multiply by 1, which gives the same result. If the odds ratio is greater than 1, for instance 2, then the intervention group has twice the odds of the control group of not smoking.

Do not overthink this. Before, we had a parameter *q* that described the probability of heads, and this was the parameter that we wished to investigate. Now we have the odds ratio, which is the quantity that we wish to investigate because we are comparing 2 coins. It is still just an unknown quantity that we wish to learn more about.

We begin by accounting for the original analysis that was done for the NEXit trial using the NHST approach, and then we shall account for a new Bayesian analysis of the data.

#### Null Hypothesis Significance Testing of the NEXit Trial

Of the 1590 participants randomly allocated into the NEXit trial, 1502 responded to follow-up regarding primary outcomes. Table 1 gives the maximum likelihood estimates for the odds ratios determined using logistic regression for the two primary outcome measures: the 95% confidence intervals and *P* values. Before the analysis, the researchers decided to perform 2-tailed tests at the .05 threshold. As Table 1 shows, the null hypothesis that the odds ratio is 1 (ie, no effect) was rejected (*P* values are <.05). Now recall that in the NHST approach, we use the maximum likelihood to *estimate* the fixed population odds ratio, and that the confidence interval should be interpreted such that the true population odds ratio lies within these limits for 95% of all the 95% confidence intervals that could be created if we were to redo the NEXit trial.

#### Bayesian Analysis of the NEXit Trial

The Bayesian approach begins by assigning prior probabilities to unknown quantities. We used flat priors for all unknown quantities, assigning equal probability to all values before seeing any data. This actually goes against our general recommendation, but we stick to flat priors so that we can defer any discussion about nonflat priors. Using Bayes’ theorem, we computed posterior distributions over the unknown quantities and then use these posterior distributions to answer questions about the quantity of interest. In this case, we care about the odds ratio comparing the intervention group with the control group.

**Figure 7:**
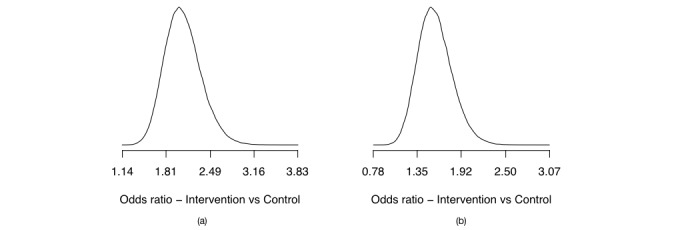
(a) NEXit trial prolonged abstinence: an approximation of the density of the posterior distribution of the odds ratio comparing intervention versus control. (b) NEXit trial point prevalence: an approximation of the density of the posterior distribution of the odds ratio comparing intervention versus control.

Figure 7 (part a) depicts the posterior distribution of the odds ratio of prolonged abstinence when comparing intervention versus control. Figure 7 (part b) similarly depicts the posterior distribution of the odds ratio of point prevalence when comparing intervention versus control.

The statistical model has done the statistical inference, and now it is up to the researcher to do the scientific inference. We have two outcome measures, which we have analyzed in terms of odds ratios. If the odds ratio is 1, then the intervention has no effect; if it is less than 1, then it has a negative effect; and if it is greater than 1, than it has a positive effect. We may therefore set up a series of questions to support our decision-making process. What is the probability that the odds ratios are greater than 1.0, 1.5, 2.0, and 2.5? The answers to these questions are given by the posterior distributions (this is why the outcome of a Bayesian analysis is the full distribution and not just a single value; we want to use the entire distribution to make a scientific inference). Table 2 summarizes the answers to these questions. As we can see, the posterior distribution tells us that it is very likely that the intervention had a positive effect on both prolonged and point prevalence outcome measures, since the posterior assigns more than 99% to these cases. It also seems more likely than not that the odds ratios for these outcomes were greater than 1.5. The odds ratio for prolonged abstinence is further more likely to be above 2.0 and, while the probability is severely lower at the 2.5 odds ratio, there is still 7.05% probability that the odds ratio is greater than 2.5.

The NEXit intervention is a fully automated intervention that does not require any interaction from health professionals. It is therefore cheap to offer and scales to large populations instantly. Participants are not put at harm and can stop the intervention at any time. It seems justifiable to offer the intervention to university students who want to quit smoking, given what the posterior distributions regarding prolonged and point prevalence abstinence tell us about the effect of NEXit. These posterior probabilities are of course calculated using a mathematical model that may or may not be a good approximation of the real world, so there is no escaping that one must assess the model chosen along with other factors. While we would like to confirm these results, and good research practice dictates that we should not blindly trust the results of a single study, if we assume that these are the only data available to us then the justification stands.

**Table 1:** Original analysis of the NEXit trial. Odds ratios compare intervention with control, given by logistic regression.

Outcome	Odds ratio	95% CI	*P* value^a^
Prolonged abstinence	2.05	1.58-2.66	≤.001
Point prevalence	1.57	1.19-2.05	.001

^a^2-tailed.

**Table 2:** Posterior probability of odds ratios at certain thresholds.

Outcome	Odds ratio			
	>1.0	>1.5	>2.0	>2.5
Prolonged abstinence	>99.99%	99.05%	57.37%	7.05%
Point prevalence	99.96%	62.50%	4.19%	0.054%

#### Comparing Null Hypothesis Significance Testing Versus the Bayesian Approach

It is actually not very easy or straightforward to compare the quantities that the NHST and the Bayesian approach produce. The numbers in Table 1 are in terms of the likelihood of the data—that is, whether the data are extreme given a hypothesized world. The numbers in Table 2 tell us the relative probability among the different worlds directly, given the data that we have collected. While the two approaches may seem to come to the same conclusion in this case—they both agree that the intervention has an effect—it is important to note that the NHST approach only says that the population effect is not 0 and has based this judgment on an arbitrarily chosen threshold, while at the same time imagining that the experiment could be repeated many times. The Bayesian approach says nothing about statistical significance, but rather communicates what we know about the NEXit intervention given the data at hand; it is the researcher’s job to transfer the statistical analysis to the real world. It is also the researcher’s job to judge the data in light of the model that was chosen, the way the data were collected, existing scientific knowledge, and the novelty of the result. Such things are not meant to be answered by statistical models.

### AMADEUS Trial

Much like the NEXit trial, the AMADEUS trial invited college and university students in Sweden to partake in the evaluation of a novel text-based alcohol intervention. The goal was to show that the intervention would reduce alcohol consumption in the group that was given access to the intervention as compared with the control group, who were referred to a website on which they could answer questions about their alcohol consumption and get feedback. The trial ran during the spring of 2016 and included participants who had at least two heavy episodic drinking occasions per month, defined as drinking more than 4 (women) or 5 (men) standard drinks on 1 occasion. The primary outcome measure was the total number of standard drinks consumed per week.

#### Statistical Model

The outcome measure in the AMADEUS trial was not a coin flip, as there are more than two possible outcomes when asking an individual how many standard drinks they consume per week. Rather, the outcome is a count variable: a variable that can take on values of 0, 1, 2, and so on (participants were not allowed to answer in partial standard drinks). To model this type of data, the researchers decided to use a negative binomial regression model. Just like the logistic regression model used for NEXit has an important quantity known as the odds ratio, the negative binomial regression has a quantity known as the *incident rate ratio* (IRR). This quantity should be interpreted as follows: take the number of standard drinks that the control group drinks on average and multiply by the IRR to get the number of standard drinks that the intervention group drinks on average. Therefore, an IRR of 1 would mean that there was no difference between the groups, less than 1 would mean that the intervention group drank less, and greater than 1 would mean that the intervention group drank more than the control group.

#### Null Hypothesis Significance Testing of the AMADEUS Trial

From the 896 randomly allocated participants, 816 responses to the primary outcome measure were collected. The IRR was determined using negative binomial regression, and a predefined threshold of .05 was used to determine statistical significance. Table 3 presents the maximum likelihood estimate of IRR, 95% confidence interval, and *P* value. The null hypothesis that the 2 groups consumed the same amount of alcohol after the intervention could not be rejected, since the IRR could not be shown to be significantly different from 1. The population value falls within 95% of all the 95% confidence intervals that can be computed.

#### Bayesian Analysis of the AMADEUS Trial

**Figure 8:**
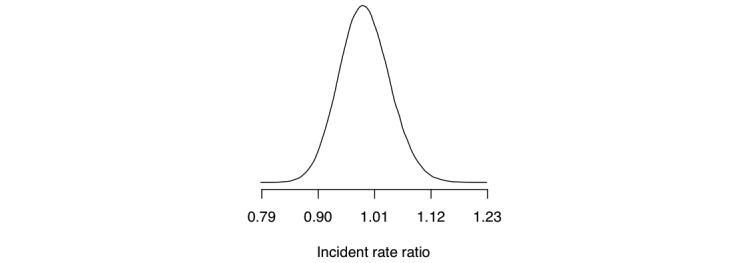
Approximation of the density of the posterior distribution of the incident rate ratio, comparing intervention versus control in the AMADEUS trial.

As we know by now, the Bayesian approach begins by assigning prior distributions to unknown quantities, and we used flat priors as before (assigning equal probability to all values of the unknown quantities before taking into account the data). Using Bayes’ theorem, we computed the posterior distribution over the IRR, depicted in Figure 8 (comparing the total weekly consumption of the intervention group with that of the control group). This is the outcome of the Bayesian analysis, and we can now use this posterior distribution to answer a series of scientific questions.

The AMADEUS trial tested a novel text-based intervention delivered to mobile phones versus referral to a website with a questionnaire and feedback. Let us assume that it was decided that there are certain levels of effect that have real-world implications. For instance, we may define a major preference for the novel intervention if the IRR is less than 0.9 (IRR<0.9), a minor preference if the IRR is between 0.9 and 1.0 (0.9<IRR<1.0), a minor preference for referring to the questionnaire and feedback if the IRR is between 1.0 and 1.1 (1.0<IRR<1.1), and a major preference if the IRR is above 1.1 (IRR>1.1). Table 4 presents these cases as questions, along with their answers. There is a small probability of a major preference for the novel intervention (the posterior probability that IRR was <0.9 is 3.3%). There is a 55.4% probability of a minor preference for the novel intervention, and a 39.6% probability of a minor preference for referring to the questionnaire. Finally, there is a 1.8% probability that referring to the questionnaire should be majorly preferred.

The routine practice at colleges and universities in Sweden is to email all students each year and refer them to the questionnaire and feedback that the control group was offered in the AMADEUS study. Should the novel intervention under trial be considered helpful and replace the questionnaire? It is interesting to note that the original publication [22] discussed potential issues with the study being underpowered and nonsignificance of the hypothesis tests, while the Bayesian approach that we have taken here allows us to discuss the real-world ramifications of the data collected. Based on the data that were collected during the trial and the model that we have chosen, we can say that it is more likely than not that the intervention had a more positive effect in the trial than referral to the questionnaire, but the difference in probability is small. We must therefore assess other factors, including an investigation into the uptake of the two different approaches: an intervention with a small effect that is used by many could be preferred to an intervention with great effect used by few. There are more information technology costs involved in the novel intervention, yet there is less administration from the student health care centers. This type of reasoning must be guided by researchers and experts, and made available to potential users and practitioners so that they take this into account before deciding whether the novel intervention is suitable.

**Table 3:** Original null hypotheses significance testing of the AMADEUS trial. Incident rate ratio (IRR) is given comparing intervention with control, as per negative binomial regression.

Outcome	IRR	95% CI	*P* value^a^
Weekly alcohol consumption	0.99	0.90-1.09	.83

^a^2-tailed.

**Table 4:** Posterior probability of incident rate ratio (IRR) for predefined effect levels.

Outcome	IRR<0.9	0.9<IRR<1.0	1.0<IRR<1.1	IRR>1.1
Weekly alcohol consumption	3.3%	55.4%	39.6%	1.8%

#### Comparing Null Hypothesis Significance Testing Versus the Bayesian Approach

The NHST analysis presented in Table 3 does not reject the null hypothesis; thus, the analysis cannot say anything about the effect of the novel intervention. Crucially, it does not tell us what the probability of the intervention having a positive or negative effect is, but rather the maximum likelihood estimate is just a sample from a sampling distribution for which we do not know the mean (the population value). It is unfortunate that, due to a conventionally decided threshold of .05 and a test against a very strict null hypothesis, the analysis leads us to a dead end from where we cannot express much more about the intervention.

The Bayesian analysis gives us a posterior distribution, and then the scientific inference can begin. Scientific inference cannot rely on conventional thresholds applied across all research fields, but rather scientific inference must be based on the real-world context and study parameters. The levels we choose to assess the effect can be understood by readers because these chosen levels have direct real-world implications—no such connection can be made to a .05 *P* value threshold.

## Discussion

### Null Hypothesis Significance Testing and Bayesian Analysis

Setting aside the mathematical differences between the two approaches, the most prominent difference is perhaps that the Bayesian approach put forward here does not incorporate the same type of null hypothesis testing that is so strongly rooted in conventional practice. This ties into the fact that the output from the Bayesian analysis is the posterior distribution over the parameters of our model. Therefore, the Bayesian approach does not attempt to identify a fixed value for the parameters and dichotomize the world into significant and nonsignificant, but rather relies on the researcher to do the scientific inference and not to delegate this obligation to the statistical model. It should not be forgotten that all statistical inference is based on a model, whether we take the NHST or Bayesian approach, and that these models are approximations of the real world. In both cases, there needs to be a leap of faith that the model chosen is a good enough approximation. We should therefore be careful not to let the model alone make assessments of the bias of the coin, but rather we must take what the model tells us and then go back to the real world and do the scientific inference ourselves.

We expect researchers to add their interpretation of their results, grounded in previous studies and current theory, balanced with cost and benefit, etc. We have purposely kept short the analyses that we have presented, but a full analysis cannot end with a posterior distribution; some scientific inference needs to be conducted. One attractive aspect of the Bayesian analyses that we have conducted herein is the way in which we ask questions of the models that have been created. For instance, the questions in Table 2 relate to the odds ratio, a quantity that can be interpreted with a real-world meaning. Compare this with an arbitrary threshold for the *P* value, which only applies in the null hypothesis world, and even then is difficult to connect to a real-world quantity.

In the NHST approach, we are assessing the population value, and we state upfront our intentions: if the null hypothesis is rejected, then we will say that the coin is biased. In this sense, we are giving a license to the statistical model to do scientific inference. Once the analysis is complete and the null hypothesis is rejected, we are not much wiser about the population value; as we have discussed, confidence intervals are not as good an indication of the location of the fixed population value as we might think. In case the hypothesis is not rejected; we have very little use of our analysis. Furthermore, the NHST approach is rooted in the idea of being able to redo the experiment many times (so as to get a sampling distribution). Even if we can rely on theoretical results to get this sampling distribution without actually going back in time and redoing the experiment, the underlying idea can be somewhat problematic. What do we mean by redoing an experiment? Can we redo a randomized controlled trial while keeping all things equal and recruiting a new sample from the study population? We might just overlook this philosophical obstacle if we like, but we should not forget that we are asking our statistical models to use such an assumption to make dichotomous decisions.

The Bayesian analysis outputs a posterior distribution, which then must be used to assess whether the coin is fair. We can say something about the value of the quantity of interest given our data, since the posterior distribution is a distribution over all possible values of the quantity. There exist Bayesian hypothesis frameworks that allow for a systematic way of making dichotomous decisions, and the interested reader may want to look into the field of decision theory, but at the end of the day the researcher must use the posterior distribution to assess the real-world implications. Imagine that we were assessing whether a medical procedure would be beneficial for a patient. We would have to weigh this probability with the risk for the patient: a 95% probability in favor of the procedure may be necessary if the procedure is invasive (eg, surgery), while a 60% probability in favor of the procedure may be okay if it simply involves a patient taking part in a seminar.

### Prior Distributions

It is usually the prior distribution that is contested by non-Bayesian proponents. How can we know anything about a parameter before we collect any data? While it is not made explicit, the non-Bayesian approach does in a sense assume flat priors on all parameters, which is why many newcomers to the Bayesian field feel that flat priors should be used all the time. However, the belief that flat priors are objective because they assign the same probability to all outcomes is not well grounded. Consider, for instance, the NEXit trial, where we used flat priors, which encodes that before we analyze the data we believe that all outcomes are equally likely. This is, however, subjective: believing it equally likely that 20% to 25% in the intervention group will quit smoking and that 90% to 95% will quit smoking. We know that brief interventions usually have a small to moderate effect size; thus, assuming a flat prior is a subjective choice going against what is known. Therefore, subjective modelling choices are unavoidable, regardless of whether one takes the Bayesian approach. The fact that the Bayesian approach requires researchers to explicitly state their prior beliefs is actually a boon, since it forces us to be explicit about this choice, rather than hiding it. Had this paper focused solely on the analysis of the NEXit and AMADEUS trials, we would have followed the suggestion of Spiegelhalter et al [23] to conduct our analysis under several priors, one that encodes indifference, one that encodes the genuine opinion among practitioners, and one that encodes skepticism toward the new intervention. It should also be noted, as McShane et al [3] pointed out, that while using a *P* value threshold may seem like a way to break subjective interpretations of statistical analyses, *P* values are highly subjective in the sense that the choice of which models to use, which covariates to include, which tests to perform, etc, all produce different *P* values.

### Interpreting Results

Practitioners, patients, the media, journal editors, and reviewers are keen to ask “does it work?” or “is it significant?” It is of course convenient to tell a patient that an intervention has been proven to have effect in a scientific study, but such statements are vague at best and lying at worst, and are still based on statistical models with arbitrarily decided-upon thresholds and null hypotheses. We should be communicating the probability that the intervention effect lies within a given range, such as that the odds ratio is greater than 1. Practitioners, patients, the media, journals, and reviewers can then use their own situation and expertise to assess the implications. We can take the posterior distribution and set it into economic and social contexts. An intervention with a 75% probability of a positive effect may still be defensible to implement, since it may be very cheap and noninvasive, while an intervention that has 95% probability of a positive effect might not be economically feasible to implement. Once we remove ourselves from the dichotomization of evidence, other things start to take precedence: critically assessing the models chosen, evaluating the quality of the data, interpreting the real-world impact of the results, etc.

We argue that the dichotomization, or be it trichotomization, is more misleading and misunderstood than Amrhein and Greenland [2] and McShane et al [3] pointed out. Many researchers and readers of scientific literature interpret statistical significance as true and nonstatistical significance as false, but this dichotomization does not exist, since statistical significance splits the world into a true state within which there exists an effect and a state in which there is ambivalence, which is not the opposite of the true state. It is not a not-true state and not a false state. Thinking in terms of statistical significance leads to a very difficult to understand dichotomization. The proposal from Benjamin et al [1] would further complicate matters, as we would end up in an even more difficult to understand trichotomization, and it raises the question of whether scientific discoveries based on *P* value thresholds of .05 from the past should now be considered nonsignificant.

### Conclusion and Call for Papers

While, compared with the NHST approach, the use of Bayesian methods to analyze randomized controlled trials is virtually nonexistent, it has increased over the past few years (Lee and Chu [24]). As further evidence of the traction Bayesian methods are achieving, the US Food and Drug Administration has released guidelines for the use of Bayesian statistics in medical device clinical trials [25].

It may yet be some time until all trials report Bayesian posteriors with scientific inference; it is nevertheless time to both educate researches about Bayesian methods and include these methods alongside current practice. The *Journal of Medical Internet Research* has issued a call for papers for a special theme issue that will be dedicated to the (re-)analysis of data from randomized controlled trials using a Bayesian framework. We invite researchers to reanalyze data from their previously published trials and write a short paper about their new analysis. Please see the call for papers on JMIR’s website (https://www.jmir.org/announcement/view/172) for further details.

## Abbreviations

IRR: incident rate ratio
NHST: null hypothesis significance testing
